# Rise in Murine Typhus in Galveston County, Texas, USA, 2018

**DOI:** 10.3201/eid2605.191505

**Published:** 2020-05

**Authors:** Karla Ruiz, Randy Valcin, Philip Keiser, Lucas S. Blanton

**Affiliations:** Galveston County Health District, Texas City, Texas, USA (K. Ruiz, R. Valcin, P. Keiser);; University of Texas Medical Branch, Galveston, Texas, USA (P. Keiser, L.S. Blanton)

**Keywords:** Rickettsia typhi, murine typhus, endemic typhus, fleaborne typhus, bacteria, Texas

## Abstract

Murine typhus, an undifferentiated febrile illness caused by *Rickettsia typhi*, is increasing in prevalence and distribution throughout Texas. In 2018, a total of 40 cases of murine typhus were reported in Galveston County. This increase, unprecedented since the 1940s, highlights the importance of awareness by physicians and public health officials.

Murine typhus is an undifferentiated febrile illness endemic worldwide in tropical and subtropical seaboard regions, where rats and rat fleas (*Xenopsylla cheopis*) are involved in the maintenance and transmission of the etiologic agent, *Rickettsia typhi* (*1*). Once prevalent in the United States, the disease was nearly eradicated following vector control practices of the 1940s using DDT (*2*). In 2012, murine typhus was identified in 2 patients from Galveston, Texas. The identification of cases in a city where murine typhus was perceived to have been eliminated prompted the investigation and identification of 12 patients from Galveston County in 2013 (*3*). Since then, murine typhus has been reported to the Galveston County Health District (GCHD) yearly (1 case in 2014, 8 in 2015, 2 in 2016, and 17 in 2017). Murine typhus has also increased in prevalence and distribution throughout Texas (*4*). To call attention to this increasingly prevalent disease, we describe an increase of murine typhus reported to the GCHD in 2018.

In Texas, murine typhus is a reportable disease. Our data were collected as part of investigatory efforts of the GCHD to document typhus group rickettsioses (TGR) in Galveston County. The case definition for confirmed TGR includes a clinically compatible illness (acute onset of fever with >1 symptoms including headache, myalgia, anorexia, rash, nausea/vomiting, thrombocytopenia, and elevated hepatic transaminases) with 1 of the following: IgG seroconversion from acute- and convalescent-phase specimens, PCR amplification of *R. typhi*, immunohistochemical demonstration of the organism within tissue specimens, or culture isolation of *R. typhi*. We defined probable cases as those having an indirect immunofluorescence assay antibody titer of >1:128 during a compatible illness.

In 2018, a total of 40 cases (12 confirmed and 28 probable) were reported to the GCHD. All cases were considered as TGRs based on serology and were identified throughout the county, affecting persons from 8 cities (Dickinson, Friendswood, Galveston, Kemah, La Marque, League City, Santa Fe, Texas City) and 2 unincorporated areas (Appendix Figure). The city of Galveston reported the most cases (11, 28%). Although 1 case occurred in January, the others occurred during April–November, with the greatest numbers in June (8, 20%) and July (10, 25%). There was a male predominance (23, 58%) and the median age was 46.5 (range 4–87) years. Frequently reported signs and symptoms included fever (40, 100%), malaise (36, 90%), myalgias (34, 85%), headache (29, 73%), anorexia (22, 55%), nausea/vomiting (22, 55%), rash (20, 50%), and elevated hepatic transaminases (17, 43%) (Table). With the exception of more nausea/vomiting in probable cases, there were no differences in signs or symptoms between confirmed and probable cases. Thirty (75%) patients were hospitalized, and 2 (5%) required intensive care. Details of treatment were known in 35 cases (34 patients received doxycycline and 1 received ciprofloxacin). No deaths were reported. Flea exposure was reported frequently (22, 55%). Patients reported a variety of exposures to mammalian hosts for fleas (Table).

**Table:** Clinical and epidemiologic features of patients with confirmed and probable murine typhus in Galveston County, Texas, 2018

Feature	No. (%) patients			p value*
	Confirmed cases, n = 12	Probable cases, n = 28	Total, n = 40	
Fever	12 (100)	28 (100)	40 (100)	0.99
Malaise	10 (83)	26 (93)	36 (90)	0.57
Myalgia	11 (92)	23 (82)	34 (85)	0.65
Headache	9 (75)	20 (71)	29 (73)	0.99
Anorexia	7 (58)	15 (54)	22 (55)	0.99
Nausea/vomiting	3 (25)	19 (68)	22 (55)	0.02
Chills	1 (8)	8 (29)	9 (23)	0.23
Abdominal pain	0	5 (18)	5 (13)	0.30
Rash	7 (58)	13 (46)	20 (50)	0.73
Hepatic transaminase elevation†	5 (42)	12 (43)	17 (43)	0.99
Thrombocytopenia‡	6 (50)	5 (18)	11 (28)	0.06
Flea exposure§	5 (42)	17 (61)	22 (55)	0.32
Opossum exposure¶	6 (50)	20 (71)	26 (65)	0.28
Dog ownership#	5 (42)	18 (64)	23 (58)	0.30
Cat ownership#	6 (50)	7 (25)	13 (33)	0.15
Stray dog exposure#	2 (17)	4 (14)	6 (15)	0.99
Stray cat exposure#	4 (33)	16 (57)	20 (50)	0.30
Rodent exposure**	4 (33)	6 (21)	10 (25)	0.45
Raccoon exposure¶	2 (17)	3 (11)	5 (13)	0.63

*Fisher exact test was performed to compare the frequency of features in those with confirmed versus probable murine typhus. With the exception of more nausea/vomiting in the probable cases group, there were no significant differences between the groups. †Defined as any aspartate aminotransferase or alanine aminotransferase level over the reference laboratories’ upper limit of normal. ‡Defined as <150,000 platelets/μL blood. §Patients were asked whether fleas were present in their environment (i.e., yard, neighborhood, house, work) or if they had been bitten by fleas in the 3 weeks before disease onset. ¶Patients were asked whether other wild animals were present in their environment (i.e., yard, neighborhood, house, work). If they answered yes, they were asked to specify the type of animal. #Patients were asked whether dogs or cats were present in their environment (i.e., yard, neighborhood, house, work). If they answered yes, they were asked whether these animals were owned or strays. **Patients were asked whether rodents were present in their environment (i.e., yard, neighborhood, house, work).

Murine typhus was once prevalent in the United States, with a peak of 5,401 cases in 1944. After eradication efforts using DDT were implemented in 1945, incidence plummeted (e.g., 98 cases in 1956) (*2*). In the decades that followed, parts of southern California and the most southern counties of Texas remained endemic. In these areas, a transmission cycle involving opossums (*Didelphis virginiana*) and cat fleas (*Ctenocephalides felis*) was suspected (*1*). After the recognized reemergence of murine typhus in Galveston, studies have demonstrated that 67% of opossums are seroreactive and 7% of *C. felis* fleas collected from these animals are infected with *R. typhi* (*5*). In contrast, only 0.3% of *C. felis* fleas collected from feral cats are infected (*6*). Although the detailed reservoir–vector dynamics involved with the increasing distribution of *R. typhi* are yet to be elucidated, the broad geographic range of opossums in North America and the ubiquity of cat fleas parasitizing opossums (*7*) cause concern for the spread of murine typhus to areas not yet endemic.

The increase in reported cases may be related not only to ecologic factors but also to increased recognition by physicians and enhanced surveillance. Indeed, murine typhus is well recognized at the University of Texas Medical Branch (UTMB), the largest healthcare system in Galveston County. Furthermore, murine typhus is now recognized as a cause of fever in local children. In a study performed at UTMB during 2012–2016, no pediatric patients had yet been identified (*8*). The later recognition in the pediatric age group was spurred in part by a pediatric case in the fall of 2017, which occurred after local education efforts. Two recently published case series of murine typhus in children may have also contributed (*9*,*10*). Finally, since September 2017, the GCHD has distributed several email advisories alerting local physicians of murine typhus.

The rise of murine typhus in Galveston is in step with the apparent increase in distribution across Texas. Awareness of this difficult to recognize undifferentiated febrile illness is paramount not only within Texas but also in other parts of the United States where opossums and cat fleas are distributed.

Appendix Map of the Galveston County, Texas area showing the distribution of murine typhus cases in 2018.

## References

[1] Azad AF, Radulovic S, Higgins JA, Noden BH, Troyer JM. Flea-borne rickettsioses: ecologic considerations. Emerg Infect Dis. 1997;3:319–27. 10.3201/eid0303.970308 9284376 PMC2627639

[2] Pratt HD. The changing picture of murine typhus in the United States. Ann N Y Acad Sci. 1958;70:516–27. 10.1111/j.1749-6632.1958.tb35408.x 13559914

[3] Blanton LS, Vohra RF, Bouyer DH, Walker DH. Reemergence of murine typhus in Galveston, Texas, USA, 2013. Emerg Infect Dis. 2015;21:484–6. 10.3201/eid2103.140716 25695758 PMC4344263

[4] Murray KO, Evert N, Mayes B, Fonken E, Erickson T, Garcia MN, et al. Typhus group rickettsiosis, Texas, USA, 2003–2013. Emerg Infect Dis. 2017;23:645–8. 10.3201/eid2304.160958 28322701 PMC5367421

[5] Blanton LS, Idowu BM, Tatsch TN, Henderson JM, Bouyer DH, Walker DH. Opossums and cat fleas: new insights in the ecology of murine typhus in Galveston, Texas. Am J Trop Med Hyg. 2016;95:457–61. 10.4269/ajtmh.16-0197 27273642 PMC4973200

[6] Blanton LS, Vohra RF, Fistein L, Quade B, Walker DH, Bouyer DH. Rickettsiae within the fleas of feral cats in Galveston, Texas. Vector Borne Zoonotic Dis. 2019;19:647–51. 10.1089/vbz.2018.2402 30835649 PMC6716191

[7] Rust MK. The biology and ecology of cat fleas and advancements in their pest management: A review. Insects. 2017;8:E118. 10.3390/insects8040118 29077073 PMC5746801

[8] Vohra RF, Walker DH, Blanton LS. Analysis of health-care charges in murine typhus: need for improved clinical recognition and diagnostics for acute disease. Am J Trop Med Hyg. 2018;98:1594–8. 10.4269/ajtmh.17-0411 29637877 PMC6086180

[9] Erickson T, da Silva J, Nolan MS, Marquez L, Munoz FM, Murray KO. Newly recognized pediatric cases of typhus group rickettsiosis, Houston, Texas, USA. Emerg Infect Dis. 2017;23:2068–71. 10.3201/eid2312.170631 29148369 PMC5708246

[10] Howard A, Fergie J. Murine typhus in South Texas children: an 18-year review. Pediatr Infect Dis J. 2018;37:1071–6. 10.1097/INF.0000000000001954 29465481

