# Supplementary material for: Rise in Murine Typhus in Galveston County, Texas, USA, 2018
Source: Emerg Infect Dis. 2020 May;26(5):1044–6. doi: 10.3201/eid2605.191505 (PMC7181902; doi:10.3201/eid2605.191505)
Supplement: Appendix — Map of the Galveston County, Texas area showing the distribution of murine typhus cases in 2018. [file 19-1505-Techapp-s1.pdf]

Article DOI: https://doi.org/10.3201/eid2605.191505

Rise in Murine Typhus in Galveston County, Texas, USA, 2018

Appendix

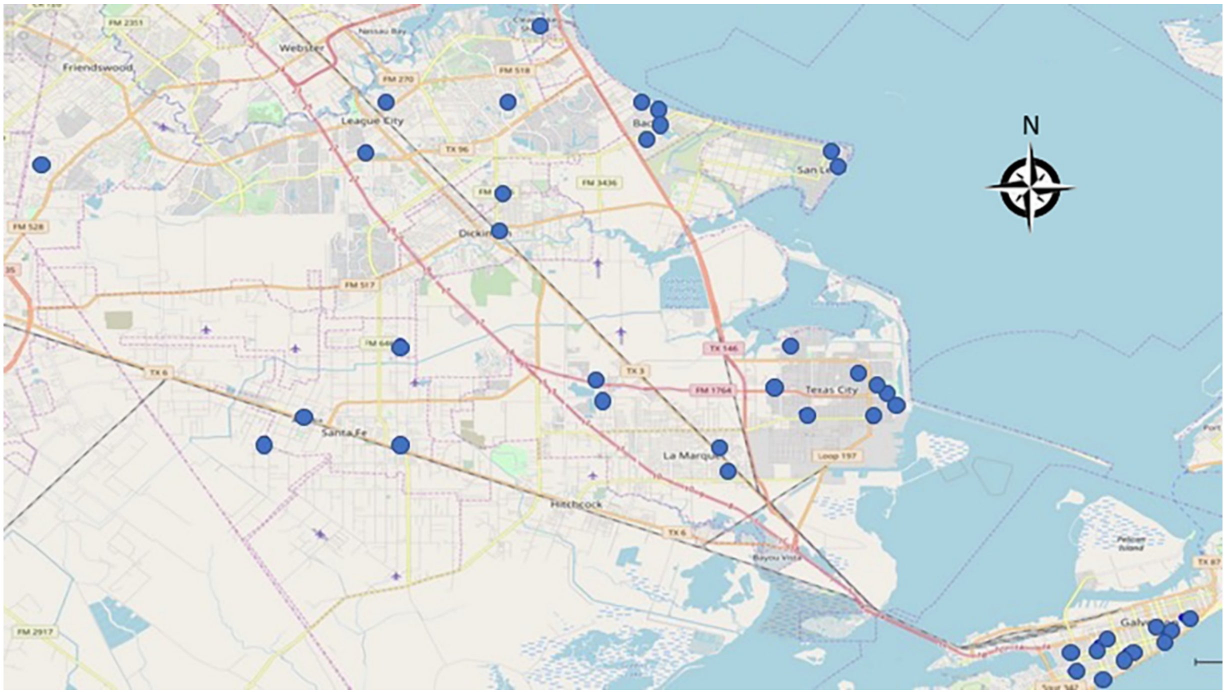

Appendix Figure. Forty cases of murine typhus (blue circles) were distributed throughout Galveston County, Texas, in 2018. There were no cases clustered within households.
